# Prescribing sodium-glucose co-transporter-2 inhibitors for type 2 diabetes in primary care: influence of renal function and heart failure diagnosis

**DOI:** 10.1186/s12933-021-01316-4

**Published:** 2021-06-28

**Authors:** William Hinton, Michael D. Feher, Neil Munro, Mark Joy, Simon de Lusignan

**Affiliations:** 1 Nuffield Department of Primary Care Health Sciences, University of Oxford, Oxford, UK; 2 Department of Clinical and Experimental Medicine, University of Surrey, Guildford, UK; 3 Royal College of General Practitioners (RCGP) Research and Surveillance Centre (RSC), London, UK

**Keywords:** Type 2, Diabetes Mellitus, Heart Failure, Kidney Function Tests, Computerized Medical Record Systems, Sodium-Glucose Transporter 2 Inhibitors

## Abstract

**Background:**

Sodium-glucose co-transporter-2 inhibitors (SGLT-2is) are licenced for initiation for glucose lowering in people with type 2 diabetes (T2DM) with an estimated glomerular filtration rate (eGFR) ≥ 60 mL/min/1.73m^2^). However, recent trial data have shown that these medications have renal and cardio-protective effects, even for impaired kidney function. The extent to which trial evidence and updated guidelines have influenced real-world prescribing of SGLT-2is is not known, particularly with co-administration of diuretics.

**Methods:**

We performed a cross-sectional analysis of people with T2DM registered with practices in the Oxford-Royal College of General Practitioners (RCGP) Research and Surveillance Centre (RSC) database on the 31st July 2019. We calculated the percentage of people prescribed SGLT-2is according to eGFR categories (< 45, 45–59, and ≥ 60 mL/min/1.73m^2^), with a heart failure diagnosis and stratified by body mass index categories (underweight, normal weight, overweight, obese), and with concomitant prescription of a diuretic. Multilevel logistic regression analysis was performed to determine whether heart failure diagnosis and renal function were associated with SGLT-2i prescribing.

**Results:**

From a population of 242,624 people with T2DM across 419 practices, 11.0% (n = 26,700) had been prescribed SGLT-2is. The majority of people initiated SGLT-2is had an eGFR ≥ 60 mL/min/1.73m^2^ (93.2%), and 4.3% had a heart failure diagnosis. 9,226 (3.8%) people were prescribed SGLT-2is as an add-on to their diuretic prescription. People in the highest eGFR category (≥ 60 mL/min/1.73m^2^) were more likely to be prescribed SGLT-2is than those in eGFR lower categories. Overweight (OR 2.05, 95% CI 1.841–2.274) and obese people (OR 3.84, 95% CI 3.472–4.250) were also more likely to be prescribed these medications, whilst use of diuretics (OR 0.74, 95% CI 0.682–0.804) and heart failure (OR 0.81, 95% CI 0.653–0.998) were associated with lower odds of being prescribed SGLT-2is.

**Conclusions:**

Prescribing patterns of SGLT-2is for glucose lowering in T2DM in primary care generally concur with licenced indications according to recommended renal thresholds. A small percentage of people with heart failure were prescribed SGLT-2is for T2DM. An updated analysis is merited should UK National Institute for Health Care and Excellence prescribing guidelines for T2DM be revised to incorporate new data on the benefits for those with reduced renal function or with heart failure.

**Supplementary Information:**

The online version contains supplementary material available at 10.1186/s12933-021-01316-4.

## Background

Sodium-glucose co-transporter-2 inhibitors (SGLT-2is) are oral glucose-lowering medications, which are now established drugs in the treatment of type 2 diabetes. They improve glycaemic control by preventing reabsorption of glucose through the proximal tubule of the kidney, and inducing glycosuria [1]. The efficacy of SGLT-2is is reduced when renal function declines; this is reflected in the current licence for initiation at an estimated glomerular filtration rate (eGFR) ≥ 60 mL/min/1.73m^2^, and discontinuation < 45 mL/min/1.73m^2^ [2]. However, recent evidence suggests that these medications have both renal and cardio-protective effects in high risk groups, including those with impaired kidney function.

Recent findings from the cardiovascular safety trials specific to SGLT-2is (EMPA-REG OUTCOME, CANVAS Progam, DECLARE-TIMI 58, and VERTIS-CV) showed that in comparison to placebo treated participants with established cardiovascular disease or at high cardiovascular risk, each drug (empagliflozin, canagliflozin, dapagliflozin, and ertugliflozin respectively) reduced the risk of hospitalisation for heart failure independently of glucose-lowering [3–6]. Moreover, renal protective effects have been demonstrated for this drug class [7–10]. The European Association for the Study of Diabetes (EASD) and American Diabetes Association (ADA) subsequently updated their guidelines for the use of antihyperglycaemic medication in type 2 diabetes to include SGLT-2is as a second-line therapy in people with a history of heart failure or chronic kidney disease [11]. The exact mechanisms behind these positive findings are yet to be determined, but it has been suggested that SGLT-2is may enhance the effect of loop-diuretics in terms of diuresis and natriuresis [12]. Currently, concomitant use of these drugs isn’t advised due to increased risk of volume depletion [13]. The extent to which trial data and guidelines have influenced prescribing in real-world clinical practice is not known, particularly when used in combination with other medications.

The following study was carried out to explore the clinical characteristics of people with type 2 diabetes prescribed SGLT-2is in an English primary care setting, and to determine whether renal function and heart failure diagnosis are associated with the likelihood of prescribing this medication in the management of type 2 diabetes.

## Methods

### Aims and objectives

The aims of this study were to identify individuals with type 2 diabetes that have previously been prescribed SGLT-2is. The objectives were to report the prevalence of people prescribed SGLT-2is according to: (1) their renal function; (2) presence of a heart failure diagnosis and body mass index category (BMI); and (3) previous prescriptions of diuretics (concurrently or ever prescribed).

We also explored whether the presence of a heart failure diagnosis and renal function are associated with SGLT-2i prescribing in people with type 2 diabetes after adjusting for known confounders.

### Study design and data source

We conducted a cross-sectional analysis using the Oxford-Royal College of General Practitioners (RCGP) Research and Surveillance Centre (RSC) database. Data were extracted for adults (≥ 18 years) with type 2 diabetes registered with an Oxford-RCGP RSC practice on the 31st July 2019. The Oxford-RCGP RSC is a primary care sentinel network of volunteer practices distributed throughout England, and comprises a nationally representative sample of patients [14].

In the UK, primary care management of type 2 diabetes is undertaken through general practices with GP and nurse support. Prescribing recommendations for the management of type 2 diabetes in the UK follows the National Institute for Health Care and Excellence (NICE) guidelines, which covers England and Wales, whilst Scotland has the Scottish Intercollegiate Guidelines Network (SIGN) guidelines, which are the recommended standards of care for primary care physicians within the National Health Service of the UK [15, 16].

UK primary care records have been computerised for over twenty years [17]; data are recorded using clinical codes and free-text. Clinical codes are derived from the Read classification [18], which includes codes for diagnoses, therapies, and processes of care. The Read classification was recently replaced by the Systematized Nomenclature of Medicine Clinical Terms (SNOMED CT) [19]. Data completeness of the Oxford-RCGP RSC database is high [14, 20], which is largely due to the Quality and Outcomes Framework, a pay-for-performance incentive scheme that has been in place since 2004, to encourage improved coding and management of chronic diseases [21]. The planned methods for this study have previously been described [22].

### Study population

Using the Oxford-RCGP RSC database, we identified people with type 2 diabetes using a comprehensive two-step ontological-based approach [20]. The first step identifies all people with diabetes using diagnostic codes, blood glucose test results, and diabetes medications. In step 2, people were categorised according to diabetes type via a seven-step algorithm that considers diagnosis codes, medications, BMI, and age at first insulin prescription.

### Renal function and heart failure

To determine renal function, we used eGFR values. The eGFR measurements were computed using a previously described ontological method, which uses a minimum of two serum creatinine values recorded at least 90 days apart [23]. Heart failure was identified by searching for codes to indicate the presence of the condition (Additional file 1: Appendix 1).

### Statistical analysis

Within the type 2 diabetes cohort, we calculated the prevalence of people with at least one previous prescription for an SGLT-2i, and compared their clinical characteristics (age, gender, ethnicity, socioeconomic status, and duration of diabetes) with the rest of the type 2 diabetes population. Ethnicity was categorised into five major groups (White, Asian, Black, Mixed, and Other), which were defined according to the official UK ethnicity categories by the Office for National Statistics [24]. An established ontological method was also applied to improve data capture for ethnicity. This takes into account recorded ethnicity and language spoken that may infer ethnicity [25]. To determine socioeconomic status, postcodes of individuals in the database were assigned Index of Multiple of Deprivation (IMD) scores, which were converted into categorical variables, quintiles that ranged from 1 (most deprived) to 5 (least deprived) [26]. This conversion process occurs at the point of data extraction, with individual postcodes subsequently removed to maintain pseudonymisation.

The subgroup of people prescribed SGLT-2is were then stratified according to their renal function, and the proportions in each eGFR category (< 45, 45–59, and ≥ 60 mL/min/1.73m^2^) were calculated. Within the same subgroup, we compared BMI categories (underweight: < 18.5 kg/m^2^; normal weight: 18.5–24.9; overweight: 25.0–29.9; obese: ≥ 30) of people with heart failure to those without. We used eGFR and BMI values closest to the first the SGLT-2i prescription. The proportion of people prescribed SGLT-2is as an add-on to diuretic therapy (loop diuretics, potassium sparing diuretics, and thiazides) or after discontinuation were also calculated. These summary statistics were reported using counts and percentages for categorical data, whilst means [with standard deviation SD)] were used to describe continuous data.

To investigate whether heart failure and renal function were associated with SGLT-2i prescribing, we performed a multilevel logistic regression analysis, with clustering to account for variability at the practice level. This was a complete case analysis. The model was adjusted for age, gender, ethnicity, IMD quintile, BMI, systolic blood pressure, glycated haemoglobin (HbA1c), presence of cardiovascular disease (defined as one or more codes for myocardial infarction, angina, atrial fibrillation, stroke, and peripheral artery disease), and use of diuretics. Exposures and covariates were defined as the closest recording (in the individual’s medical record) up to two years after diagnosis of diabetes. Individuals that had previously been prescribed an SGLT-2i within two years after diagnosis of diabetes were excluded from this analysis. Odds ratios (OR) with 95% confidence intervals (CI), and *P* values were reported for each exposure variable. All analyses were performed in R statistical software version 3.5.3.

### Sensitivity analysis

As a sensitivity analysis, we re-ran the multilevel logistic regression model after performing multiple imputation. Due to the complexities of trying to impute missing data for ethnicity (data that are not missing at random in primary care) [27], we assigned these data to the white ethnic category. We used predictive mean matching for our imputation method for ten simulated models. The coefficient estimates were then pooled according to Rubin’s rules [28].

## Results

The Oxford-RCGP RSC network comprised 3,372,309 adults across 419 practices at the time of data extraction (31st July 2019). Within this population, we identified 242,624 (7.2%) people with type 2 diabetes. Of these, 26,700 (11.0%) had previously been prescribed at least one SGLT-2i. People prescribed SGLT-2is were younger compared to those not prescribed this drug (Table 1), but a higher proportion had been living with diabetes for five or more years (72.8 vs. 66.6%).

**Table 1:** Clinical characteristics of people with type 2 diabetes with or without a prescription of SGLT-2i

Characteristic	T2DM prescribed SGLT-2i (N = 26,700)	T2DM not prescribed SGLT-2i (N = 215,924)
Age (years)	59.8 ± 10.9	67.5 ± 13.9
Male	15,680 (58.7)	118,958 (55.1)
Ethnicity		
White	17,735 (66.4)	142,664 (66.1)
Asian	3369 (12.6)	23,559 (10.9)
Black	804 (3.0)	8924 (4.1)
Mixed	210 (0.8)	1752 (0.8)
Other	279 (1.0)	2090 (1.0)
Missing	4303 (16.1)	36,935 (17.1)
IMD quintile		
IMD quintile 5 (least deprived)	4984 (18.7)	44,284 (20.5)
IMD quintile 4	5249 (19.7)	44,301 (20.5)
IMD quintile 3	5063 (19.0)	41,355 (19.2)
IMD quintile 2	4873 (18.3)	38,448 (17.8)
IMD quintile 1 (most deprived)	5774 (21.6)	41,227 (19.1)
Missing	757 (2.8)	6309 (2.9)
Duration of diabetes (years)	9.4 ± 6.3	9.3 ± 7.3
< 1	1582 (5.9)	16,019 (7.4)
1–4	5686 (21.3)	56,009 (25.9)
5–9	8187 (30.7)	58,595 (27.1)
≥ 10	11,245 (42.1)	85,301 (39.5)

Data are presented as n (%) or mean (± SD)

*IMD* Index of Multiple Deprivation, *SGLT-2i* sodium–glucose co‐transporter‐2 inhibitor, *T2DM* Type 2 diabetes

The vast majority of people initiated SGLT-2is had an eGFR ≥ 60 mL/min/1.73m^2^ (93.2%), whilst only 463 (1.7%) people had an eGFR < 60 at initiation (Table 2). 1157 (4.3%) people prescribed SGLT-2is with type 2 diabetes had heart failure; the majority of this group were overweight or obese (Table 3). Approximately two fifths of people with type 2 diabetes were prescribed diuretics (n = 104,524; 43.1%), whilst 9226 (3.8%) people were initiated on an SGLT-2i as an add-on therapy to their diuretic prescription.

**Table 2:** SGLT-2i prescriptions in type 2 diabetes according to renal function (eGFR) categories

eGFR category^a^	Prescribed SGLT-2i (N = 26,700)
< 45	56 (0.2)
45–59	407 (1.5)
≥ 60	24,895 (93.2)
Missing	1342 (5.0)

Data are presented as n (%)

*eGFR* estimated glomerular filtration rate, *SGLT-2i* sodium–glucose co‐transporter‐2 inhibitor

^a^eGFR closest to first SGLT-2i prescription

**Table 3:** SGLT-2i prescriptions in type 2 diabetes according to BMI category in individuals with or without heart failure

BMI category^a^	SGLT-2i with heart failure (N = 1157)	SGLT-2i without heart failure (N = 25,543)
Underweight	0 (0.0)	18 (0.1)
Normal weight	54 (4.7)	1396 (5.5)
Overweight	229 (19.8)	6149 (24.1)
Obese	855 (73.9)	17,595 (68.9)
Missing	19 (1.6)	385 (1.5)

Data are presented as n(%)

*BMI* body mass index, *SGLT-2i* sodium–glucose co‐transporter‐2 inhibitor

^a^BMI categories closest to first SGLT-2- prescription: underweight, < 18.5 kg/m^2^; normal, 18.5 − 24.9 kg/m^2^; overweight, 25.0 − 29.9 kg/m^2^; obese, > 30 kg/m^2^

In the multilevel logistic regression analysis (N = 239,719), SGLT-2is were more likely to be prescribed in males than females (Table 4). The odds of being prescribed this drug increased by 1.03 (95% CI 1.029–1.031; *p* < 0.001) per unit increase in HbA1c (mmol/mol). Similarly, as BMI increased, the likelihood of being prescribed SGLT-2is grew; the OR was lower for people in the underweight category compared to those of normal weight, but higher in those that were overweight, and higher still, in those in the obese category (Table 4 and Additional file 2: Figure S1).

**Table 4:** Prescribing of SGLT-2is in type 2 diabetes; multilevel logistic regression model (clustered at the practice level)

Characteristic	OR	95% CI	*p*-value
Age (years)	0.98	0.975–0.979	< 0.001
Gender			
Female	1.00 [Reference]		
Male	1.13	1.083–1.185	< 0.001
Ethnicity			
White	1.00 [Reference]		
Asian	0.88	0.806–0.954	0.002
Black	0.60	0.522–0.683	< 0.001
Mixed	0.66	0.521–0.843	< 0.001
Other	0.75	0.597–0.939	0.012
IMD Quintile			
1 (most deprived)	0.95	0.871–1.044	0.300
2	0.99	0.907–1.071	0.737
3	1.00	0.922–1.079	0.953
4	1.01	0.937–1.088	0.795
5 (least deprived)	1.00 [Reference]		
BMI category (kg/m^2^)^a^			
Underweight	0.21	0.083–0.549	0.001
Normal	1.00 [Reference]		
Overweight	2.05	1.841–2.274	< 0.001
Obese	3.84	3.472–4.250	< 0.001
eGFR (mL/min/1.73m^2^)			
< 45	0.03	0.011–0.080	< 0.001
45–59	0.18	0.145–0.224	< 0.001
≥ 60	1.00 [Reference]		
Comorbidities			
Heart failure	0.81	0.653–0.998	0.048
CVD	0.69	0.636–0.739	< 0.001
Other covariates			
Systolic BP (mmHg)	1.00	0.996–0.999	0.001
HbA1c (mmol/mol)	1.03	1.029–1.031	< 0.001
Diuretic	0.74	0.682–0.804	< 0.001

*OR* odds ratio, *BMI* body mass index, *BP* blood pressure, *CVD* cardiovascular disease, *HbA1c* glycated haemoglobin, *IMD* Index of Multiple Deprivation

^a^BMI categories closest to first SGLT-2- prescription: underweight, < 18.5 kg/m^2^; normal, 18.5–24.9 kg/m^2^; overweight, 25.0–29.9 kg/m^2^; obese, ≥ 30 kg/m^2^

In terms of renal function, people with an eGFR < 60 mL/min/1.73m^2^ were less likely to be prescribed SGLT-2is than those with an eGFR ≥ 60 mL/min/1.73m^2^. The odds of being prescribed SGLT-2is were lower in people with heart failure, albeit this association was only just significant (OR 0.81, 95% CI 0.653–0.998; p = 0.048). The presence of cardiovascular disease was also associated with reduced odds of being prescribed SGLT-2is, as were increasing age, use of diuretics, and being of Asian or Black ethnicity compared to White ethnicity (Table 4 and Additional file 2: Figure S1). No association was found between socioeconomic status (IMD Quintile) and SGLT-2i prescribing, and there did not appear to be a clear association for systolic blood pressure (OR 1.00, 95% CI 0.996–0.999; *p* = 0.001).

The sensitivity analysis of the imputed data reflected these findings, although people of Asian ethnicity had higher odds of being prescribed SGLT-2is than people of White ethnicity (Additional file 2: Table S1; Figure S2).

## Discussion

This cross-sectional study explored the clinical characteristics of people with type 2 diabetes in an English primary care setting, with a focus on renal function and heart failure as factors associated with a prescription for SGLT-2i. Our findings demonstrated that SGLT-2is are prescribed in about one in ten people with type 2 diabetes. In the vast majority of cases, SGLT-2is were prescribed in people with an eGFR ≥ 60 mL/min/1.73m^2^, whilst around a twentieth of this cohort had heart failure and they were mostly overweight or obese. The characteristics of people prescribed these drugs were similar to the wider type 2 diabetes population, but they were younger (by ~ 8 years) and had a longer duration of diabetes (duration ≥ 5 years: 72.8 vs 66.6%). In addition, individuals were less likely to be prescribed an SGLT-2i if they had previously been prescribed a diuretic.

High BMI appears to be a key driver for SGLT-2i prescribing in people with type 2 diabetes regardless of the presence of heart failure. The weight lowering properties of these drugs are well known, since calories are lost in the glucose when excreted in the urine, and reflect change in body weight [29]. Results from meta-analyses of pooled data from clinical trials demonstrated that participants treated with SGLT-2is lost significantly more weight than those treated with placebo or other glucose-lowering drugs (metformin, sulphonylures, and dipeptidyl peptidase 4 inhibitors) [30, 31]. Our findings therefore, reflect that clinicians are prescribing according to these established findings.

Similarly, renal function influenced propensity to prescribe SGLT-2is. The current prescribing recommendations for all SGLT-2is are determined by renal function categories of eGFR based upon registration trial data. There has been some evidence indicating that low eGFR thresholds are linked to reduced glucose lowering efficacy [32], which further supports the location of action of these drugs at the renal tubule level. Again, our results reflect that prescribing in real-world clinical practice is in accordance with guidelines. However, data from recent drug safety trials suggest that people with impaired renal function may benefit from SGLT-2i therapy.

In the SGLT-2i cardiovascular outcome trials, exploratory analyses of people with established cardiovascular disease or at high cardiovascular risk, showed that SGLT-2is delayed the progression of kidney disease and renal events compared to placebo in people with an eGFR as low as 30 mL/min/1.73m^2^ [4, 5, 8–10]. More recently, the Canagliflozin and Renal Events in Diabetes with Established Nephropathy Clinical Evaluation (CREDENCE) trial demonstrated that compared to placebo, canagliflozin reduced the relative risk of kidney failure by 30% in participants with comorbid chronic kidney disease and type 2 diabetes [33]. In response to these findings, the licence for canagliflozin was recently updated for people with diabetic kidney disease, with drug prescribing at an eGFR ≥ 30 mL/min/1.73m^2^ [34, 35]. Whilst other SGLT-2is are not currently licensed for initiation in people with an eGFR < 60 mL/min/1.73m^2^ and for continuation in those with an eGFR < 45 mL/min/1.73m^2^ [2], the emerging evidence that SGLT-2is have reno-protective effects in people impaired renal function implies that these drugs can be used to treat people with chronic kidney disease [36].

When considering the presence of heart failure in people prescribed SGLT-2is, this was lower than the prevalence of heart failure in the broader type 2 diabetes population (4.3 and 8.1% respectively). Moreover, our analysis showed that heart failure was associated with reduced likelihood of being prescribed SGLT-2is. This finding is slightly surprising given that each SGLT-2i drug reduced the risk of heart failure events compared to placebo-controlled participants in the cardiovascular outcome trials [3–6]. Yet this may reflect the earlier recommendations with these drugs to use with caution when in combination with diuretics, whereby the risk of dehydration was promoted early in the introduction of the drugs into clinical practice [37]. Our results appear to support this notion, since prescribing of diuretics was also associated with reduced odds of being prescribed SGLT-2is. However, this might be a temporal effect as the benefits within the heart failure group have only recently been incorporated into international (EASD/ADA) guidelines and have yet to be included as part of the treatment algorithm in the UK based NICE guidelines for type 2 diabetes, which focus principally on improving glycaemic control [11, 15]. There is accumulating data on the benefits of SGLT-2is on cardiac function, vascular endothelial function, and cardio-metabolic risk factors [38–43], which may further enhance utilisation of these drugs in real-world clinical practice.

Although current guidelines advise against co-administration SGLT-2is and diuretics to avoid volume depletion [13], proof of concept studies have inferred a possible synergy between the SGLT-2is and diuretics in the management of heart failure in people with type 2 diabetes [12, 44]. For example, in a randomised, open-label, parallel group study of 42 healthy participants treated with once-daily bumenatide, dapagliflozin, or both agents for seven days, followed by both agents over eight days, it was found that sodium excretion was greater when the agents were used in combination than individually. The authors concluded that use of SGLT-2is might be beneficial for use in heart failure, particularly in those with loop diuretic resistance. Despite these potential benefits, it is unknown whether there is any difference in side-effects that may require hospital admission, when taking concomitant diuretic therapy, and whether dual prescribing confers additional heart failure benefits in real-world practice. Further clinical trials are needed to help answer these questions.

### Strengths and limitations

The large sample size and wide coverage across England are key strengths of the Oxford-RCGP RSC database. Other benefits of the database include high data quality dating back to 2004, which make it an ideal resource for longitudinal follow-up of patient populations. The Oxford-RCGP RSC network also comprises a broadly representative population in terms of age, sex, and ethnicity compared to England and Wales Census data, although the more deprived population are slightly underrepresented [14]. Additional limitations relate to the secondary use of routinely collected data.

As with all observational data, the Oxford-RCGP RSC database was affected to an extent by missing data. For example, within the computerised medical records, confirmed diagnosed conditions such as heart failure, were indicated by a date field. For analytical purposes, everyone without a date was assumed not to have the condition, when in fact some cases may have had undiagnosed heart failure. In addition, key information about a patient is often recorded in the GP’s notes; data that we did not have access to for these analyses.

For other variables, we were able to use multiple imputation to impute the missing cases in our sensitivity analysis. For the most part, this confirmed the main findings for the analysis of complete cases; the finding that people with Asian ethnicity had higher odds of being prescribed SGLT-2is than White people was most likely due to assigning missing data to the White category.

## Conclusions

Prescribing for glucose lowering of SGLT-2is in type 2 diabetes in primary care concur with the licenced indications according to renal function. A history of heart failure diagnosis has not been incorporated into NICE diabetes management guidelines and current practice reflects this with the low prescription rates in heart failure in type 2 diabetes. It is worth revisiting this analysis again should NICE guidelines be updated to incorporate new data on the benefits of SGLT-2is for those with reduced renal function or with heart failure.

## Supplementary Information

**Additional file 1**: **Appendix 1**. 5-byte version 2 Read and CTV3 codes used to identify the presence of heart failure.

**Additional file 2**: **Figure S1**. A forest plot of odds ratios for characteristics associated with prescribing of SGLT-2is in people with type 2 diabetes: complete cases. **Figure S2**. A forest plot of odds ratios for characteristics associated with prescribing of SGLT-2is in people with type 2 diabetes: imputed dataset. **Table S1**. Prescribing of SGLT-2is in people with type 2 diabetes; multilevel logistic regression model of imputed dataset.

## Data Availability

The Oxford-RCGP RSC data set can be accessed by researchers; approval is given on a project-by-project basis. Researchers wishing to directly analyse the patient-level pseudonymised data are required to complete information governance training and work on the data from the secure servers at the University of Oxford. Patient-level data cannot be taken out of the secure network.

## Abbreviations

ADA: American Diabetes Association
BMI: Body mass index
BP: Blood pressure
CANVAS: Canagliflozin Cardiovascular Assessment Study
CREDENCE: Canagliflozin and Renal Events in Diabetes with Established Nephropathy Clinical Evaluation
CVD: Cardiovascular disease
DECLARE-TIMI 58: Dapagliflozin Effect on Cardiovascular Events–Thrombolysis in Myocardial Infarction 58
EASD: European Association for the Study of Diabetes
eGFR: Estimated glomerular filtration rate
EMPA-REG OUTCOME: Empagliflozin Cardiovascular Outcome Event Trial in Type 2 Diabetes Mellitus Patients–Removing Excess Glucose
HbA1c: Glycated haemoglobin
IMD: Index of Multiple Deprivation
NICE: National Institute of for Health Care and Excellence
RCGP RSC: Royal College of General Practitioners Research and Surveillance Centre
SGLT-2i: Sodium-glucose co-transporter-2 inhibitor
SIGN: Scottish Intercollegiate Guidelines Network
SNOMED CT: Systematized Nomenclature of Medicine Clinical Terms
VERTIS-CV: EValuation of ERTugliflozin effIcacy and Safety CardioVascular outcomes trial
