# Supplementary material for: Prescribing sodium-glucose co-transporter-2 inhibitors for type 2 diabetes in primary care: influence of renal function and heart failure diagnosis
Source: Cardiovasc Diabetol. 2021 Jun 28;20:130. doi: 10.1186/s12933-021-01316-4 (PMC8237469; doi:10.1186/s12933-021-01316-4)
Supplement: Supplementary file 1 — Additional file 1: Appendix 1. 5-byte version 2 Read and CTV3 codes used to identify the presence of heart failure. [file 12933_2021_1316_MOESM1_ESM.docx]

**Appendix 1.** 5-byte version 2 Read and CTV3 codes used to identify the presence of heart failure.

| **Coding System** | **Read Code** | **Description** |
| --- | --- | --- |
| Version 2 | 14A6. | H/O: heart failure |
| Version 2 | 14AM. | H/O: Heart failure in last year |
| Version 2 | 14S3. | H/O: heart recipient |
| Version 2 | 14T7. | H/O: artificial heart |
| Version 2 | 1O1.. | Heart failure confirmed |
| Version 2 | 662f. | New York Heart Association classification - class I |
| Version 2 | 662g. | New York Heart Association classification - class II |
| Version 2 | 662h. | New York Heart Association classification - class III |
| Version 2 | 662i. | New York Heart Association classification - class IV |
| Version 2 | 662p. | Heart failure 6 month review |
| Version 2 | 662T. | Congestive heart failure monitoring |
| Version 2 | 662W. | Heart failure annual review |
| Version 2 | 679W1 | Education about deteriorating heart failure |
| Version 2 | 679X. | Heart failure education |
| Version 2 | 67D4. | Heart failure information given to patient |
| Version 2 | 7901. | Other transplantation of heart |
| Version 2 | 79010 | Allotransplantation of heart NEC |
| Version 2 | 79011 | Xenotransplantation of heart |
| Version 2 | 79013 | Piggyback transplantation of heart |
| Version 2 | 79014 | Revision of implantation of prosthetic heart |
| Version 2 | 79015 | Revision of transplantation of heart NEC |
| Version 2 | 7901y | Other specified other transplantation of heart |
| Version 2 | 7901z | Other transplantation of heart NOS |
| Version 2 | 7933. | Transluminal heart assist operations |
| Version 2 | 79330 | Transluminal insertion of pulsation balloon into aorta |
| Version 2 | 79331 | Transluminal insertion of heart assist system NEC |
| Version 2 | 79332 | Transluminal maintenance of heart assist system |
| Version 2 | 79334 | Implantation of ventricular assist device |
| Version 2 | 79335 | Implantation of right ventricular assist device |
| Version 2 | 79336 | Implantation of left ventricular assist device |
| Version 2 | 79337 | Implantation of biventricular assist device |
| Version 2 | 7933y | Other specified transluminal heart assist operation |
| Version 2 | 7933z | Transluminal heart assist operation NOS |
| Version 2 | 7936J | Implantation of intravenous biventricular cardiac pacemaker system |
| Version 2 | 79379 | Implantation of biventricular cardiac pacemaker system |
| Version 2 | 793L. | Open heart assist operations |
| Version 2 | 793L0 | Open implantation of ventricular assist device |
| Version 2 | 793Ly | Other specified open heart assist operations |
| Version 2 | 793Lz | Open heart assist operations NOS |
| Version 2 | 8B29. | Cardiac failure therapy |
| Version 2 | 8CeC. | Preferred place of care for next exacerbation of heart failure |
| Version 2 | 8CL3. | Heart failure care plan discussed with patient |
| Version 2 | 8CMK. | Has heart failure management plan |
| Version 2 | 8CMW8 | Heart failure clinical pathway |
| Version 2 | 8H2S. | Admit heart failure emergency |
| Version 2 | 8HBE. | Heart failure follow-up |
| Version 2 | 8HHb. | Referral to heart failure nurse |
| Version 2 | 8HHz. | Referral to heart failure exercise programme |
| Version 2 | 8Hk0. | Referred to heart failure education group |
| Version 2 | 8HTL. | Referral to heart failure clinic |
| Version 2 | 8HTL0 | Referral to rapid access heart failure clinic |
| Version 2 | 9N0k. | Seen in heart failure clinic |
| Version 2 | 9N2p. | Seen by community heart failure nurse |
| Version 2 | 9Or0. | Heart failure review completed |
| Version 2 | G1yz1 | Rheumatic left ventricular failure |
| Version 2 | G2101 | Malignant hypertensive heart disease with congestive cardiac failure |
| Version 2 | G2111 | Benign hypertensive heart disease with congestive cardiac failure |
| Version 2 | G21z1 | Hypertensive heart disease NOS with congestive cardiac failure |
| Version 2 | G232. | Hypertensive heart and renal disease with (congestive) heart failure |
| Version 2 | G234. | Hypertensive heart and renal disease with both (congestive) heart failure and renal failure |
| Version 2 | G58.. | Heart failure |
| Version 2 | G580. | Congestive heart failure |
| Version 2 | G5800 | Acute congestive heart failure |
| Version 2 | G5801 | Chronic congestive heart failure |
| Version 2 | G5802 | Decompensated cardiac failure |
| Version 2 | G5803 | Compensated cardiac failure |
| Version 2 | G5804 | Congestive heart failure due to valvular disease |
| Version 2 | G581. | Left ventricular failure |
| Version 2 | G5810 | Acute left ventricular failure |
| Version 2 | G582. | Acute heart failure |
| Version 2 | G583. | Heart failure with normal ejection fraction |
| Version 2 | G584. | Right ventricular failure |
| Version 2 | G58z. | Heart failure NOS |
| Version 2 | Q48y1 | Congenital cardiac failure |
| Version 2 | SP084 | Heart transplant failure and rejection |
| Version 2 | TB000 | Transplantation of heart as the cause of abnormal reaction of patient, or of later complication, without mention of misadventure at the time of operation |
| Version 2 | ZV421 | [V]Heart transplanted |
| Version 2 | ZV45M | [V]Biventricular pacemaker in situ |
| CTV3 | 14A6. | H/O: heart failure |
| CTV3 | 14S3. | H/O: heart recipient |
| CTV3 | 14S4. | H/O: heart valve recipient |
| CTV3 | 14T3. | H/O: artificial heart valve |
| CTV3 | 14T7. | H/O: artificial heart |
| CTV3 | 7901. | Other transplantation of heart |
| CTV3 | 79010 | Allotransplantation of heart NEC |
| CTV3 | 79011 | Xenotransplant of heart |
| CTV3 | 79013 | Heterotopic allotransplant of heart |
| CTV3 | 79014 | Revision of implantation of prosthetic heart |
| CTV3 | 79015 | Revision of transplantation of heart NEC |
| CTV3 | 7901y | Other specified other transplantation of heart |
| CTV3 | 7901z | Other transplantation of heart NOS |
| CTV3 | 7933. | Transluminal heart assist operations |
| CTV3 | 79330 | Transluminal insertion of aortic balloon pump |
| CTV3 | 79331 | Transluminal insertion of heart assist system NEC |
| CTV3 | 79332 | Transluminal maintenance of heart assist system |
| CTV3 | 79333 | Transluminal removal of heart assist system |
| CTV3 | 7933y | Other specified transluminal heart assist operation |
| CTV3 | 7933z | Transluminal heart assist operation NOS |
| CTV3 | 793L0 | Open implantation of ventricular assist device |
| CTV3 | bm... | Vasodilators in heart failure [no drugs here] |
| CTV3 | G1yz1 | Rheumatic left ventricular failure |
| CTV3 | G2100 | Malignant hypertensive heart disease without congestive cardiac failure |
| CTV3 | G2101 | Malignant hypertensive heart disease with congestive cardiac failure |
| CTV3 | G2110 | Benign hypertensive heart disease without congestive cardiac failure |
| CTV3 | G2111 | Benign hypertensive heart disease with congestive cardiac failure |
| CTV3 | G21z1 | Hypertensive heart disease NOS with congestive cardiac failure |
| CTV3 | G232. | Hypertensive heart and renal disease with (congestive) heart failure |
| CTV3 | G233. | Hypertensive heart and renal disease with renal failure |
| CTV3 | G234. | Hypertensive heart and renal disease with both (congestive) heart failure and renal failure |
| CTV3 | G58.. | Heart failure |
| CTV3 | G5800 | Acute congestive heart failure |
| CTV3 | G5801 | Chronic congestive heart failure |
| CTV3 | G5802 | Decompensated cardiac failure |
| CTV3 | G5803 | Compensated cardiac failure |
| CTV3 | G5810 | Acute left ventricular failure |
| CTV3 | G582. | Acute heart failure |
| CTV3 | G5y4. | Post cardiac operation functional disturbance |
| CTV3 | G5y4z | Post cardiac operation heart failure NOS |
| CTV3 | H584. | Acute pulmonary oedema unspecified |
| CTV3 | H584z | Acute pulmonary oedema NOS |
| CTV3 | Q48y1 | Congenital cardiac failure |
| CTV3 | SP084 | Heart transplant failure and rejection |
| CTV3 | TA26. | Failure of sterile precautions during heart catheterisation |
| CTV3 | TA45. | Mechanical failure of instrument or apparatus during heart catheterisation |
| CTV3 | TB000 | Transplantation of heart as the cause of abnormal reaction of patient, or of later complication, without mention of misadventure at the time of operation |
| CTV3 | U6125 | [X]Failure of sterile precautions during heart catheterisation |
| CTV3 | X00xv | Implantation of ventricular assist device |
| CTV3 | X00xw | Implantation of right ventricular assist device |
| CTV3 | X00xx | Implantation of left ventricular assist device |
| CTV3 | X00xy | Implantation of biventricular assist device |
| CTV3 | X102h | Fluid overload pulmonary oedema |
| CTV3 | X102X | Acute pulmonary oedema |
| CTV3 | X102Y | Acute cardiac pulmonary oedema |
| CTV3 | X102Z | Neurogenic pulmonary oedema |
| CTV3 | X202k | Heart failure as a complication of care |
| CTV3 | X202l | Right ventricular failure |
| CTV3 | XaaH6 | Referral to rapid access heart failure clinic |
| CTV3 | XaBLt | Cardiac failure therapy |
| CTV3 | XabM9 | On optimal heart failure therapy |
| CTV3 | XaBwi | H/O: Heart failure in last year |
| CTV3 | XaBYA | Transluminal right ventricular biopsy after heart transplant |
| CTV3 | XaEgY | Refractory heart failure |
| CTV3 | XaIIU | Congestive heart failure monitoring |
| CTV3 | XaIkr | Suspected heart failure |
| CTV3 | XaILD | Referral to heart failure clinic |
| CTV3 | XaImX | Heart failure excluded |
| CTV3 | XaIpn | Heart failure confirmed |
| CTV3 | XaIPz | Heart failure screen |
| CTV3 | XaIQM | Heart failure follow-up |
| CTV3 | XaIQN | Heart failure annual review |
| CTV3 | XaJ9G | New York Heart Association classification - class I |
| CTV3 | XaJ9H | New York Heart Association classification - class II |
| CTV3 | XaJ9I | New York Heart Association classification - class III |
| CTV3 | XaJ9J | New York Heart Association classification - class IV |
| CTV3 | XaKNa | Seen by community heart failure nurse |
| CTV3 | XaKNN | Seen in heart failure clinic |
| CTV3 | XaKNW | Admit heart failure emergency |
| CTV3 | XaKNX | Referral to heart failure nurse |
| CTV3 | XaLCj | Referred by heart failure nurse specialist |
| CTV3 | XaLCm | Discharge from practice nurse heart failure clinic |
| CTV3 | XaLGJ | Did not attend practice nurse heart failure clinic |
| CTV3 | XaLMw | Heart failure information given to patient |
| CTV3 | XaLMx | Referral to heart failure exercise programme |
| CTV3 | XaLN6 | Heart failure monitoring administration |
| CTV3 | XaLN7 | Heart failure review completed |
| CTV3 | XaLNA | Heart failure care plan discussed with patient |
| CTV3 | XaLon | Heart failure 6 month review |
| CTV3 | XaLSI | Referred to heart failure education group |
| CTV3 | XaM9t | Open heart assist operations |
| CTV3 | XaM9u | Open implantation of ventricular assist device |
| CTV3 | XaM9w | Other specified open heart assist operations |
| CTV3 | XaM9x | Open heart assist operations NOS |
| CTV3 | XaMHD | Did not attend heart failure clinic |
| CTV3 | XaMKH | Implantation of intravenous biventricular cardiac pacemaker system |
| CTV3 | XaMKK | Implantation of biventricular cardiac pacemaker system |
| CTV3 | XaNUf | Heart failure education |
| CTV3 | XaO5n | Congestive heart failure due to valvular disease |
| CTV3 | XaP9J | Congestive heart failure, hypertension, age, diabetes, stroke 2 risk score |
| CTV3 | XaPiK | High risk of heart failure screening invitation |
| CTV3 | XaR8o | Heart failure resolved |
| CTV3 | XaWyi | Heart failure with normal ejection fraction |
| CTV3 | XaXBj | Fast track heart failure referral for transthoracic two dimensional echocardiogram |
| CTV3 | XaXgq | Referral to heart failure exercise programme declined |
| CTV3 | XaXgs | Referral to heart failure exercise programme not indicated |
| CTV3 | XaXIR | Referral to heart failure education group declined |
| CTV3 | XaXkR | Has heart failure management plan |
| CTV3 | XaXNh | Discharge from heart failure nurse service |
| CTV3 | XaXzw | Preferred place of care for next exacerbation of heart failure |
| CTV3 | XaY6i | Congestive heart failure, hypertension, age 2, diabetes mellitus, stroke 2, vascular disease, age, sex category score |
| CTV3 | XaYbC | Heart failure self-management plan agreed |
| CTV3 | XaYft | Education about deteriorating heart failure |
| CTV3 | XaYZN | Heart failure self-management plan review |
| CTV3 | XaZIC | Rehabilitation for heart failure |
| CTV3 | XaZih | Heart failure clinical pathway |
| CTV3 | XE0Ue | Hypertensive heart disease NOS without congestive cardiac failure |
| CTV3 | XE0V8 | Biventricular failure |
| CTV3 | XE0V9 | Heart failure NOS |
| CTV3 | XE2QG | Left ventricular failure |
| CTV3 | XM1Qn | Impaired left ventricular function |
| CTV3 | ZV421 | [V]Heart transplanted |
| CTV3 | ZV422 | [V]Heart valve transplanted |
| CTV3 | ZV45M | [V]Biventricular pacemaker in situ |
