# Supplementary material for: Prescribing sodium-glucose co-transporter-2 inhibitors for type 2 diabetes in primary care: influence of renal function and heart failure diagnosis
Source: Cardiovasc Diabetol. 2021 Jun 28;20:130. doi: 10.1186/s12933-021-01316-4 (PMC8237469; doi:10.1186/s12933-021-01316-4)

**Figure S1.** A forest plot of odds ratios for characteristics associated with prescribing of SGLT-2is in people with type 2 diabetes: complete cases

# **Supplementary file**


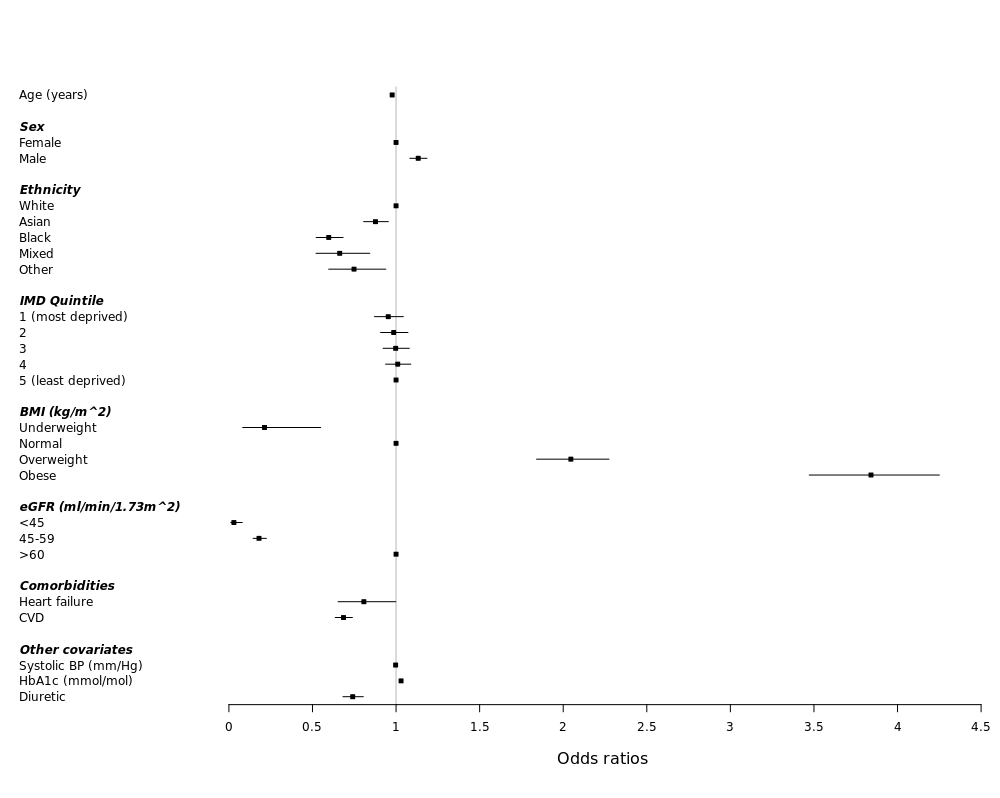


**Table S1.** Prescribing of SGLT-2is in people with type 2 diabetes; multilevel logistic regression model of imputed dataset

| **Characteristic** | **OR** | **95% CI** | ***p*-value** |
| --- | --- | --- | --- |
| Age (years) | 0.98 | 0.977 - 0.979 | <0.001 |
| **Sex** |  |  |  |
| Female | 1.00 [Reference] | | |
| Male | 1.13 | 1.093 - 1.159 | <0.001 |
| **Ethnicity** |  |  |  |
| White | 1.00 [Reference] | | |
| Asian | 1.11 | 1.043 - 1.170 | <0.001 |
| Black | 0.73 | 0.666 - 0.800 | <0.001 |
| Mixed | 0.76 | 0.639 - 0.893 | <0.001 |
| Other | 0.86 | 0.738 - 0.994 | 0.041 |
| **IMD Quintile** |  |  |  |
| 1 (most deprived) | 0.97 | 0.916 - 1.036 | 0.406 |
| 2 | 1.00 | 0.942 - 1.053 | 0.892 |
| 3 | 1.01 | 0.954 - 1.061 | 0.834 |
| 4 | 1.01 | 0.962 - 1.062 | 0.669 |
| 5 (least deprived) | 1.00 [Reference] | | |
| **BMI category (kg/m^2^)**† |  |  |  |
| Underweight | 0.62 | 0.408 - 0.956 | 0.030 |
| Normal | 1.00 [Reference] | | |
| Overweight | 1.75 | 1.631 - 1.870 | <0.001 |
| Obese | 3.42 | 3.196 - 3.658 | <0.001 |
| **eGFR (ml/min/1.73m^2^)** |  |  |  |
| <45 | 0.11 | 0.076 - 0.169 | <0.001 |
| 45-59 | 0.23 | 0.202 - 0.270 | <0.001 |
| >60 | 1.00 [Reference] | | |
| **Comorbidites** |  |  |  |
| Heart failure | 0.74 | 0.631 - 0.862 | <0.001 |
| CVD |  |  |  |
| **Other covariates** |  |  |  |
| Systolic BP (mmHg) | 1.00 | 0.997 - 0.999 | <0.001 |
| HbA1c (mmol/mol) | 1.03 | 1.027 - 1.029 | <0.001 |
| Diuretic | 0.69 | 0.646 - 0.727 | <0.001 |

OR: odds ratio. BMI, body mass index; BP: blood pressure; CVD: cardiovascular disease. HbA1c, glycated haemoglobin; IMD, Index of Multiple Deprivation..

†BMI categories closest to first SGLT2- prescription: underweight, <18.5 kg/m^2^; normal, 18.5−24.9 kg/m^2^; overweight, 25.0−29.9 kg/m^2^; obese, >30 kg/m^2^

**Figure S2.** A forest plot of odds ratios for characteristics associated with prescribing of SGLT-2is in people with type 2 diabetes: imputed dataset


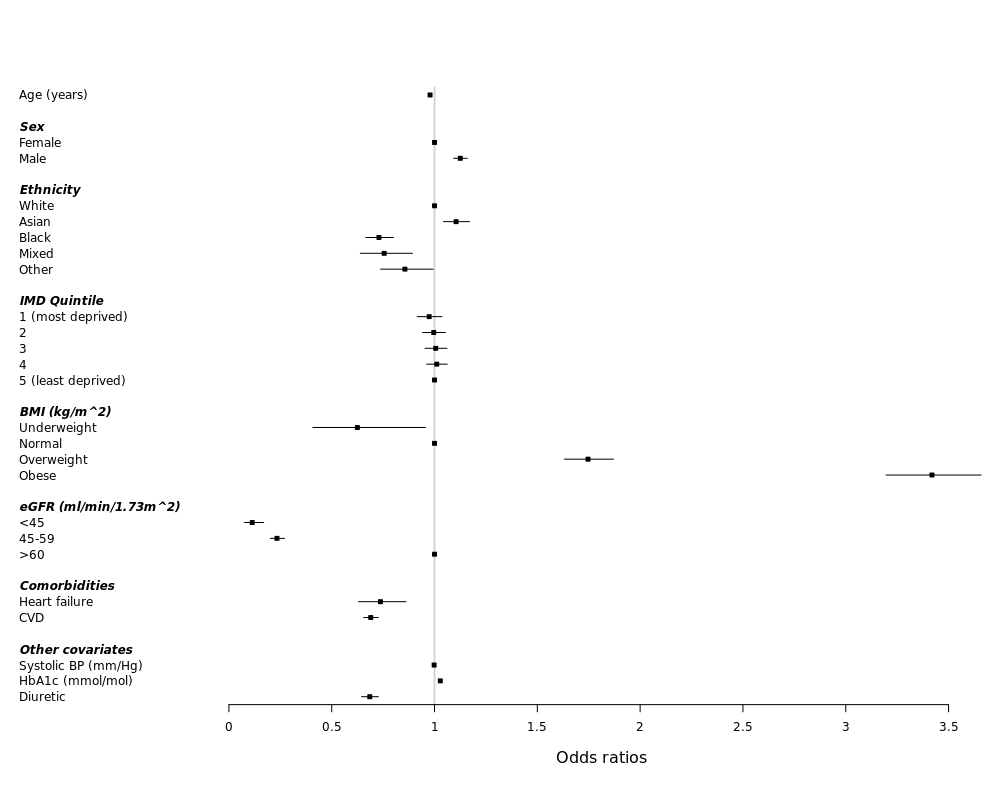

Supplement: Supplementary file 2 — Additional file 2: Figure S1. A forest plot of odds ratios for characteristics associated with prescribing of SGLT-2is in people with type 2 diabetes: complete cases. Figure S2. A forest plot of odds ratios for characteristics associated with prescribing of SGLT-2is in people with type 2 diabetes: imputed dataset. Table S1. Prescribing of SGLT-2is in people with type 2 diabetes; multilevel logistic regression model of imputed dataset. [file 12933_2021_1316_MOESM2_ESM.docx]
